# Physiological Responses of Fishes to Nutrition Management and Environmental Stresses

**DOI:** 10.3390/ani16111572

**Published:** 2026-05-22

**Authors:** László Ardó, Janka Nagyné Biró

**Affiliations:** Research Centre for Fisheries and Aquaculture, Institute of Aquaculture and Environmental Safety, Hungarian University of Agriculture and Life Sciences, Anna-liget u. 35, H-5540 Szarvas, Hungary

## Abstract

The goal of this Special Issue was to collect the latest research results on the topics of (1) the physiological response of fish to various stressful situations and nutritional changes, (2) the replacement of fish meal and fish oil with sustainable, alternative protein and lipid sources in fish feeds, and (3) supplementing fish feeds with various additives in order to enhance the immune response and increase the stress and disease resistance of fish reared in intensive systems. These topics are very important for the development of a more effective and sustainable intensive aquaculture, as fish farming systems are becoming more intensive and industrialized, which results in a more stressful environment for farmed fish. Another important challenge is providing enough high-quality fish feed to fulfill the increasing demand of intensive aquaculture. There are 14 original research articles published in this Special Issue. The authors come from a wide array of countries, and they worked with a wide variety of freshwater and marine fish species, which are important for intensive aquaculture. These articles represent only a modest contribution to the overall literature of stress and nutritional physiology of farmed fish. However, each of these papers contain interesting new information regarding the solution of the two major problems of intensifying aquaculture: environmental stresses and nutrition management.

Aquaculture industry is becoming more and more important source of fish and other kinds of seafood, while capture fish industry is stagnating or declining, because of the exploitation of wild stocks. Global aquaculture production reached 130.9 million tons in 2022, surpassing capture fisheries for the first time in history, and the role of aquaculture in fish production is projected to increase further [1]. Due to the increasing demand, fish farming systems are becoming more intensive and industrialized [2,3], which results in a more stressful environment for the farmed fish [4,5,6]. Most important environmental factors during intensive rearing of fish include stocking density, dissolved oxygen concentration, noise, temperature, pH, salinity and lighting [7]. Changes of these parameters that exceed the natural regulatory capacity of a given species cause stress, which can result in detrimental effects on welfare, growth, immune response, disease resistance and survival [4,6,8]. Better understanding the physiological mechanisms behind the stress response of fish can lead to a more effective and sustainable intensive aquaculture; therefore, this topic is an important area of today’s fisheries and aquaculture research.

Another important topic is providing enough high-quality fish feed to fulfill the increasing demand of intensive aquaculture [9,10]. Traditionally, fish meal and fish oil are the main protein and lipid sources of artificial fish feeds [11]. However, due to the limited marine resources, their replacement with components of terrestrial origin (e.g. plant or insect) is one of the key challenges for sustainability of intensive fish production [12,13]. These alternative feed ingredients must have the same or better nutritional values as fish meal and fish oil, without compromising physiological functions like immune response or liver antioxidant systems [14,15]. These physiological functions can be improved by mixing special additives into the feeds. The resulting functional fish feeds can be applied to increase the resistance of fish against infectious diseases and the numerous stressors related to intensive rearing conditions [16,17,18,19].

The goal of this Special Issue was to collect the latest research results on the topics of (1) the physiological response of fish to various stressful situations and nutritional changes, (2) replacement of fish meal and fish oil with sustainable, alternative protein and lipid sources in fish feeds, and (3) supplementing fish feeds with various additives in order to enhance the immune response and increase the stress and disease resistance of fish reared in intensive systems. There are 14 papers published in this Special Issue, and all of them are original research articles, which proves the importance and actuality of these topics. The authors come from a wide array of countries, including China, Italy, Taiwan, Russia, Greece, Iran, Hungary and the Republic of Korea. They worked with a wide variety of freshwater and marine fish species, which are important for intensive aquaculture, like black carp (*Mylopharyngodon piceus*), grass carp (*Ctenopharyngodon idella*), gibel carp (*Carassius auratus gibelio*), sea bass (*Dicentrarchus labrax*), gilthead sea bream (*Sparus aurata*), rainbow trout (*Oncorhynchus mykiss*), Atlantic salmon (*Salmo salar*), cherry salmon (*Oncorhynchus masou*), Asian sea bass (*Lates calcarifer*), mandarin fish (*Siniperca chuatsi*), African catfish (*Clarias gariepinus*), orange-spotted grouper (*Epinephelus coioides*) and American eel (*Anguilla rostrata*).

Out of the 14 published articles, two deal with the physiological mechanisms behind stress response and nutritional changes. Li et al. cloned and characterized the coding sequence of GPR84, a G-protein coupled receptor gene from grass carp. This receptor is activated by medium-chain fatty acids and is involved in inflammatory response in macrophages. They demonstrated that GPR84 in grass carp is mainly expressed in liver and spleen, and is responsive to medium-chain fatty acids, like in mammals and amphibians. In the other study, Yu et al. characterized the prolyl hydroxylase domain protein 2 (PHD2) in mandarin fish. This molecule regulates the stability of hypoxia inducible factor 1α (HIF-1α) via hydroxylation. Under hypoxia, the catalytic activity of these proteins is inhibited by the lack of oxygen, resulting in the accumulation of HIF-1α, which activates the HIF-1 regulatory pathway. In mandarin fish, PHD2 was expressed in all the tissues tested and was more highly expressed in blood and liver than in other tissues, and its overexpression could inhibit the HIF-1 pathway. Lee and Balasubramanian studied the effects of four rearing temperatures on growth, feed utilization, stress and haemato-immune responses of juvenile cherry salmon. They found 10 and 14 °C as optimal for this species, regarding growth and feeding performance.

As sustainable feed ingredients, insect and poultry by-product meals were studied as plant protein replacements by Donadelli et al. They demonstrated that sea bream adapted well to the new feed ingredients; however, these diets caused altered lipid metabolism and liver steatosis in sea bass, resulting in an increased risk to liver health. Feed ingredients of insect origin were also studied by Gebremichael et al., who replaced fish meal with black soldier fly and mealworm meals in African catfish diets. Black soldier fly meal could be used as a replacement, without any negative effects on growth, health status or feed utilization. In contrast, mealworm meal caused growth reduction and health issues. Soybean meal has been widely used as an alternative to fish meal. However, its application has limitations, as it was demonstrated by Yin et al., who found that higher doses of glycinin (a soybean antigen protein) reduced growth and caused liver and intestinal health problems in orange-spotted grouper. Rapeseed oil is a potential replacement of fish oil as a lipid source in fish feeds. Liu et al. demonstrated that high levels of erucic acid present in rapeseed oil decreased immune response and antioxidant capacity and caused lipid deposition in black carp, which means that the dose of rapeseed oil in fish feed requires optimization. Lee et al. studied the potential use of low-lipid rainbow trout feeds in rearing of parr-stage Atlantic salmons. These feeds could be used without any negative effects on growth, hematological, histological and immunological traits, compared to commercial salmon feeds. Lu et al. found that up to 20% of white fish meal could be replaced with cheaper, low-quality brown fish meal in the diet of American eels without adversely affecting growth or intestinal health, if some functional additives are also included in the feeds.

Various feed additives as components of functional fish feeds have been studied by the authors of this Special Issue. Fokina et al. demonstrated that feed supplemented with an antioxidant (dihydroquercetin) and a probiotic (arabinogalactan) of natural origin made rainbow trouts more tolerant to the summer temperature rise, resulting in lower mortality, better growth and a higher n-3 to n-6 ratio of unsaturated fatty acids. In the experiment of Akram et al., grape extract in an optimal dose of 2% enhanced cold stress tolerance, growth performance and antioxidant capacity of Asian sea bass. Yousefi et al. studied the effects of thymol, an antioxidant of plant origin on juvenile rainbow trout, and found that this feed additive in lower (100 mg/kg) dose could enhance antioxidant capacity, reduce lipid peroxidation and mortality after heat stress. However, thymol had no effect on growth or humoral immune response. A reduced positive effect of *Artemisia arborescens* supplementation on growth, biochemical, oxidative stress and immunological parameters of gilthead sea bream was observed by Tzortzatos et al., suggesting that feed additives of plant origin should be used in fish species with caution. Similarly, Wang et al. found that lower (0.01–0.02%) levels of ferroporphyrin supplementation improved the immune response of gibel carp, without affecting antioxidant and oxygen-carrying capacity, while higher doses decreased these parameters.

Research articles published in this Special Issue represent only a modest contribution to the overall literature of stress and nutritional physiology of farmed fish. However, each of these papers contain interesting new information regarding the solution of the two major problems of intensifying aquaculture: environmental stresses and nutrition management. We are grateful to the editors of Animals for their generous support, and to all the authors and external reviewers for their contributions to this Special Issue.
